# Whole genome sequencing reveals high-resolution epidemiological links between clinical and environmental *Klebsiella pneumoniae*

**DOI:** 10.1186/s13073-017-0397-1

**Published:** 2017-01-24

**Authors:** Chakkaphan Runcharoen, Danesh Moradigaravand, Beth Blane, Suporn Paksanont, Jeeranan Thammachote, Suthatip Anun, Julian Parkhill, Narisara Chantratita, Sharon J. Peacock

**Affiliations:** 1 Department of Microbiology and Immunology, Faculty of Tropical Medicine, Mahidol University, Bangkok, 10400 Thailand; 2 Wellcome Trust Sanger Institute, Wellcome Genome Campus, Hinxton, Cambridgeshire UK; 3 Department of Medicine, University of Cambridge, Addenbrooke’s Hospital, Box 157, Hills Road, Cambridge, CB2 0QQ UK; 4 Division of Clinical Microbiology, Medical Technology Department, Buddhasothorn hospital, Chachoengsao, 24000 Thailand; 5 Mahidol-Oxford Tropical Medicine Research Unit, Faculty of Tropical Medicine, Mahidol University, Bangkok, 10400 Thailand; 6 London School of Hygiene and Tropical Medicine, London, WC1E 7HT UK

**Keywords:** *Klebsiella pneumoniae*, Whole genome sequencing, Antibiotic resistance, Genomic epidemiology

## Abstract

**Background:**

*Klebsiella pneumoniae* is a gram-negative bacterial species capable of occupying a broad range of environmental and clinical habitats. Known as an opportunistic pathogen, it has recently become a major causative agent of clinical infections worldwide. Despite growing knowledge about the highly diverse population of *K. pneumoniae*, the evolution and clinical significance of environmental *K. pneumoniae*, as well as the relationship between clinical and environmental *K. pneumoniae*, are poorly defined.

**Methods:**

We isolated and sequenced *K. pneumoniae* from in-patients in a single hospital in Thailand, as well as hospital sewage, and surrounding canals and farms within a 20-km radius.

**Results:**

Phylogenetic analysis of 77 *K. pneumoniae* (48 clinical and 29 non-clinical isolates) demonstrated that the two groups were intermixed throughout the tree and in some cases resided in the same clade, suggesting recent divergence from a common ancestor. Phylogenetic comparison of the 77 Thai genomes with 286 *K. pneumoniae* from a global collection showed that Thai isolates were closely related to the clinical sub-population of the global collection, indicating that Thai clinical isolates belonged to globally circulating lineages. Dating of four Thai *K. pneumoniae* clades indicated that they emerged between 50 and 150 years ago. Despite their phylogenetic relatedness, virulence factors and β-lactamase resistance genes were more numerous in clinical than in environmental isolates. Our results indicate that clinical and environmental *K. pneumoniae* are closely related, but that hospitals may select for isolates with a more resistant and virulent genotype.

**Conclusions:**

These findings highlight the clinical relevance of environmental *K. pneumoniae* isolates.

**Electronic supplementary material:**

The online version of this article (doi:10.1186/s13073-017-0397-1) contains supplementary material, which is available to authorized users.

## Background

*Klebsiella pneumoniae* is a clinically important gram-negative bacterium associated with opportunistic infection in patients with a compromised immune system or receiving other forms of complex medical care [1–3]. This species is disseminated in healthcare settings via person-to-person contact, medical equipment, and contaminated environments [2, 3]. The emergence of multidrug-resistant *K. pneumoniae* is becoming an increasingly serious issue for clinical practice, largely related at the present time to isolates that express extended-spectrum β-lactamase (ESBL) enzymes that hydrolyze a broad spectrum of β-lactams [4, 5]. This is compounded by the emergence of *K. pneumoniae* that express carbapenemases such as KPC-type β-lactamase [5–7] and the recent detection of colistin-resistant *K. pneumoniae* due to the presence of the *mcr-1* gene [8]. These resistance genes are carried on mobile genetic elements that facilitate their spread within and between bacterial species, a process that is likely to result in a rise in the number of *K. pneumoniae* infections that are very difficult to treat. Furthermore, multidrug-resistant *K. pneumoniae* are associated with nosocomial outbreaks, particularly in high prevalence countries including those in East Asia [9, 10].

Beyond healthcare settings and hospital patients, *K. pneumoniae* is ubiquitous in nature and occupies a diverse range of niches. These include environmental sources, such as soil and wastewater, mucosal surfaces and the gut of humans and animals, and food sources, such as meat [11]. Environmental *K. pneumoniae* is less well studied than isolate collections associated with clinical disease. Some studies have shown that *K. pneumoniae* of environmental origin are highly similar to clinical isolates with regards to phenotypic and some genetic features [12–14], but others have reported differences in virulence characteristics between the two groups [15, 16]. The parallel evolution of *K. pneumoniae* and putative acquisition of antimicrobial resistance determinants and virulence factors in healthcare settings and the environment have led non-clinical habitats to be considered as potential reservoirs for hyper-virulent and hyper-resistant *K. pneumoniae* [15], although evidence to support the potential clinical importance of non-clinical *K. pneumoniae* is inconclusive. A recent study of the clinical relevance of meat-source *K. pneumoniae* showed differences in antibiotic resistance but similar virulence characteristics for isolates from retail meat and those associated with urinary tract infections in humans [16].

Here, we report the findings of an in-depth comparison of the evolution and epidemiology of ESBL-positive *K. pneumoniae* using a One Health approach [17, 18]. We utilized the fine-scale resolution of whole genome sequencing to investigate genetic relatedness, antimicrobial resistance, and the presence of genes encoding virulence determinants in an unbiased, prospective collection of *K. pneumoniae* from patients in one hospital, as well as from environmental water, hospital sewage, and farm waste in the proximity of the hospital. These genomes were placed into a global context through a comparison with isolates recovered from various clinical and non-clinical sources worldwide [19]. Our results highlight the clinical relevance of environmental *K. pneumoniae* isolates, and demonstrate that environmental and clinical *K. pneumoniae* are highly related but that hospitals select for *K. pneumoniae* with a more antimicrobial-resistant and virulent genotype.

## Methods

### Study design and bacterial isolates

The bacterial collection consisted of 77 ESBL-producing *K. pneumoniae* isolated between 2014 and 2015. Clinical isolates (*n* = 48) were obtained from consecutive patients with positive samples processed by the diagnostic microbiology laboratory at Bhuddhasothorn Hospital, Chachoengsao, Bangkok, Thailand between December 2014 and April 2015. Data were collected on date of isolation and sample type, and samples were de-duplicated so that only one isolate per patient was included. Speciation and ESBL positivity were initially determined using Standard Operating Procedures supplied by the Department of Medical Science, Ministry of Public Heath, Thailand and Clinical and Laboratory Standards Institute (CLSI) guidelines (M100-S24 and M100-S25), respectively. The species was subsequently confirmed using matrix-assisted laser desorption/ionization time-of-flight mass spectrometry (MALDI-TOF MS; Biotyper version 3.1, Bruker Daltonics, Coventry, UK). Antimicrobial susceptibility testing was repeated using the N206 card on the Vitek 2 instrument (bioMérieux, Marcy l’Étoile, France) calibrated against EUCAST breakpoints, and these results were used during the analysis.

Environmental and livestock-associated isolates (*n* = 29) were obtained through a cross-sectional survey between January 2015 and February 2015. Wastewater samples were collected from 27 canals and 11 farms within a 20-km radius of Bhuddhasothorn Hospital. The farms reared pigs (*n* = 2), chickens (*n* = 6), ducks (*n* = 2), and both chickens and ducks (*n* = 1). Samples were collected from wastewater collection areas (commonly concrete gullies that drained waste from animal housing). The longitude and latitude of each sampling site were recorded using GPSMAP 60CSx (Garmin, Taiwan). A further two wastewater samples were taken from the Bhuddhasothorn Hospital wastewater treatment system (one pre-treatment and one post-treatment water sample). At each site, grab samples of 0.5 L each were collected into sterile bottles containing 9 mg of sodium thiosulfate pentahydrate (Merck, Darmstadt, Germany). All samples were transported to the laboratory on ice packs in the dark and processed within 12 h.

One mL of triplicate serial 10-fold dilutions of wastewater samples were concentrated using the filtration technique onto 0.45-μm pore size filter membranes (Merck, Darmstadt, Germany). Membranes were then placed onto the surface of ESBL Brilliance agar (Oxoid, Basingstoke, UK) and incubated for 48 h at 35 °C in air. For each sampling site, up to 10 colonies suspected to be *K. pneumoniae* based on color (green) were picked and the presence of ESBL confirmed using the combination disc test (M100-S24 and M100-S25). *Escherichia coli* ATCC25922 and *K. pneumoniae* ATCC700603 were used as negative and positive controls, respectively. Positive colonies were speciated using MALDI-TOF MS, and antimicrobial susceptibility testing of confirmed *K. pneumoniae* was determined using the N206 card as described above. All isolates were stored at −80 °C until further analysis.

### Whole genome sequencing and pan-genome analysis

DNA extraction, sequencing, and assembly of reads were performed as previously described [20]. Sequencing was performed on an Illumina HiSeq2000. Details of reads, depth of coverage, and N50 are provided in Additional file 1. The sequence reads were submitted to the European Nucleotide Archive (ENA) under accession numbers [ENA:ERP012787 and ENA:PRJEB11403]. An average coverage of 85-fold was achieved. Genomes were assembled using Velvet [21] with the pipeline and improvements found at https://github.com/sanger-pathogens/vr-codebase and https://github.com/sanger-pathogens/assembly_improvement. The de novo assemblies were annotated using Prokka [22], and sequence types (STs) were identified from the sequence data using a multilocus sequence typing (MLST) database (http://bigsdb.pasteur.fr/klebsiella/) and an in-house script (https://github.com/sanger-pathogens/mlst_check).

### Phylogenetic analysis and substitution rate calculation

Study genomes were contextualized against a global collection. Sequence data for 286 *K. pneumoniae* isolates reported previously [19] were downloaded from the ENA and combined with the 77 study genomes. As in our study, these isolates were also sequenced on a HiSeq sequencing system. Short reads for the 363 isolates were mapped against the reference genome *K. pneumoniae* Ecl8 (accession number: HF536482 CANH01000000) using SMALT v0.7.4 (http://www.sanger.ac.uk/science/tools/smalt-0). An in-house tool that combined SAMtools mpileup [23] and BCFtools, as detailed in [24], was used to annotate single nucleotide polymorphisms (SNPs), after which the pairwise SNP distances were calculated from the multiple alignment to obtain the phylogenetic tree. Mapping each genome to the reference genome allowed us to identify genes that were conserved in the core genome of the study isolates and reference genome. We constructed a maximum likelihood tree using FastTree version 2.1.3 with 100 bootstraps and a midpoint root [25]. We employed FigTree (http://tree.bio.ed.ac.uk/software/*figtree*/), Microreact (www.microreact.org), and in-house tools to visualize the results.

To determine the substitution rate for each clade (see the Results section for more details), reads were first mapped within each clade to the sequence obtained after concatenating contigs for the isolate with the best contigs statistics, i.e., the isolate with the highest N50 value. After mapping the reads to the new references, high-density SNP regions (which are indicative of putative recombination events) were removed from the multiple alignments using Gubbins, which works best for closely related isolates and detects high SNP density regions based on a significantly higher number of variable sites in a sliding window across the genome compared to the rest of the genome [26]. The significance of the temporal signal was assessed according the *R*-squared value obtained from the root-to-tip distance versus time of isolation plot. We generated 10,000 sample sets by bootstrapping and assessed the value of *R*-squared against the distribution of *R*-squared values. Of four clades we identified on the phylogenetic tree of the Thai and global isolates, we found strong signals (>99%) for clades 1 and 3, after leaving one isolate out from either clade. BEAST version 1.7 was used to estimate the substitution rate and date the phylogenetic tree for the clades with significant temporal signal [27], using a strict molecular clock and a lognormal and uniform prior distribution models for base frequencies with constant population size. Three chains of BEAST were run for 50 million generations (sampling every 10 generations) and checked as to whether the runs had converged on similar values. Convergence was controlled using effective sample size (ESS) value (we considered a cut-off of 200 ESS for convergence). We excluded 10 million initial steps as a burn-in phase and merged the out trees with the Tree-Annotator program in the BEAST package, and chose the model in which convergence always occurred (for the clades we tested here, the strict molecular clock with a uniform prior distribution for base frequencies always converged).

### Identification of antimicrobial resistance determinants, virulence factors, and plasmids

We employed the srst2 package [28], which takes short reads and maps them to reference sequences, to find antibiotic resistance genes, virulence factors, and plasmid replicons. The sequences for 79 virulence genes were obtained from the Pasteur institute data repository (http://bigsdb.web.pasteur.fr/klebsiella/klebsiella.html, http://bigsdb.pasteur.fr/perl/bigsdb/bigsdb.pl?db=pubmlst_klebsiella_seqdef_public&page=downloadAlleles). An in-house tool was used to visualize the results. To find the possible context of the virulence (i.e., chromosomal or plasmid based), we first found the contig in which the virulence factor was located and then extracted the 5-kb sequences upstream and downstream of the gene; if this exceeded the contig size, we considered the end and start of the contig. We then performed blast searches using the NCBI non-redundant nucleotide database to find out whether the hit sequences corresponded to a chromosomal or a plasmid region. Furthermore, we assessed the significance between the presence of virulence factors in the clinical/environmental sub-populations by performing logistic regression. To this end, we took the presence/absence of individual genes as the categorical predictor parameter and the environmental/clinical status as the categorical dependent variable. Significance of association (*p* value < 0.05) was then assessed by considering the z-statistic, which is the regression coefficient divided by its standard error and has a standard normal distribution.

## Results

**Fig. 1:**
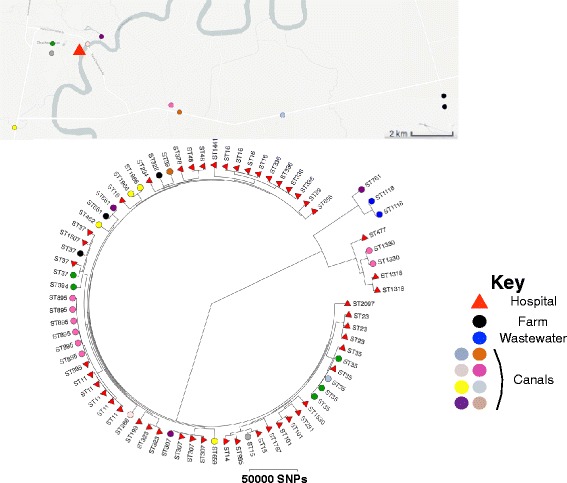
Map showing the geographical origin of study isolates. The *triangle* denotes Bhuddhasothorn Hospital and the maximum likelihood tree for 77 *K. pneumoniae* isolates from clinical and environmental samples (canals, livestock, and hospital sewage) showing the distribution of STs across the population. *Triangle* and *circles* correspond to clinical and environmental isolates, respectively. Wastewater isolates were recovered from the hospital and therefore have the same location as the hospital in the map. An interactive map can be found at www.microreact.org/project/Skog9F1ex

A One Health approach was taken for the sampling and isolation of ESBL-positive *K. pneumoniae* from clinical samples in a hospital in Thailand, together with hospital sewage, environmental (canal) water, and wastewater from farms within a 20-km radius of the hospital. A phylogenetic tree of the whole genomes of the 77 *K. pneumoniae* isolates (48 clinical and 29 environmental, of which 24 were from canals, 3 from farms, and 2 from untreated hospital sewage) revealed a diverse population containing three major lineages, one of which contained the majority of isolates (Fig. 1). Several minor clades were also apparent, some of which showed evidence of recent expansion (Fig. 1). Clinical and environmental isolates were intermixed throughout the tree and in some cases resided in the same clade, suggesting recent divergence from a common ancestor (Fig. 1). The 3 isolates of farm origin were distributed across the tree, and clustered with canal and clinical isolates or with canal isolates alone (Fig. 1). High genetic diversity was also reflected by the number of STs (*n* = 38) to which the 77 isolates were assigned. This included STs that have been isolated elsewhere in the world. For example, ST35, ST307, and ST16 were recently recovered from clinical settings in France, the USA, and the Netherlands, respectively [29–31]. Mapping the geographical distribution of isolates recovered from the environment demonstrated that isolates from the same sampling site were frequently very genetically diverse (Fig. 1).

**Fig. 2:**
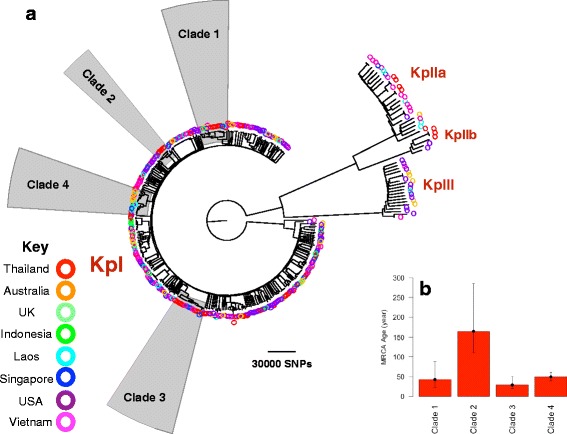
**a** Phylogenetic tree of the 77 Thai isolates placed in the context of a global collection. Each *color* corresponds to a country. *Shaded* clades refer to those described in the text. **b** Dating the most recent common ancestor for clades identified on the phylogenetic tree. To calculate the mean and upper and lower bounds of age root for clades 1 and 3, which lacked a temporal signal, we divided the root-to-tip distances of the root by mean substitution rates (and upper and lower bounds for 95% confidence interval) of clades 1 and 3

To further characterize the 77 study genomes, we combined them with genomes for a global collection of 286 *K. pneumoniae complex* isolates [19]. The resulting phylogenetic tree revealed that Thai isolates were dispersed across the combined population. Isolates in the global collection had been defined previously based on phylogroup, and Thai isolates were observed to cluster in phylogroups KpI, KpIIa, and KpIIb, with the majority residing in KpI (Fig. 2a). This broader genetic context highlighted the presence of several clades of Thai isolates; those containing at least 4 Thai isolates (clades 1 to 4) were subjected to more detailed analysis to uncover their recent evolutionary history. These clades included 30 of the 77 total isolates. A strong temporal signal was found for clade 1 and clade 3 after removing regions of recombination. This allowed us to estimate the substitution rates, which were 8.33 × 10^−7^ (95% confidence interval [CI]: 6.23 × 10^−7^, 1.05 × 10^−6^) per site per year for clade 1 and 3.78 × 10^−7^ (95% CI: 7.47 × 10^−8^, 7.54 × 10^−7^) per site per year for clade 3. The most recent common ancestor (MRCA) for clades 1 and 3 was estimated to exist 50 to 70 years ago (Fig. 2b). Using the average substitution rates for clades 1 and 3, we also estimated the age of the MRCA for clades 2 and 4 to be 150 and 50 years ago, respectively (Fig. 2b). We conclude that expansion of these *K. pneumoniae* clones has taken place over the past few decades.

**Fig. 3:**
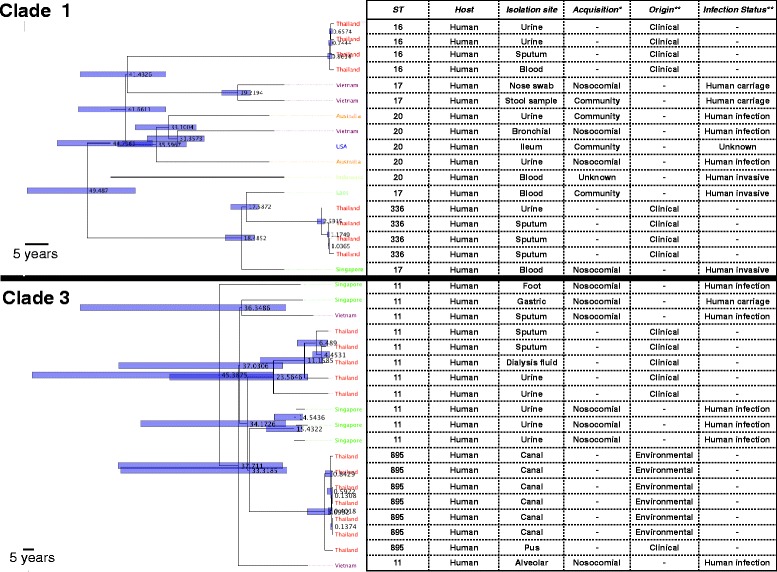
Dated trees for clades with a temporal signal. The *numbers* on the nodes signify the node age (in years), and the *bars* show 95% confidence intervals. The symbols * and ** signify the specific features of the Thai and the global collection, respectively

Thai isolates residing in clades 1 and 3 were intermixed with isolates from other countries in the global collection (Fig. 3). The Thai isolates in clade 1 fell into two sub-clades of clinical origin, both of which were estimated to have emerged in the past few decades. These were more distantly related to two clinical isolates from community and nosocomial infection in the global collection (Fig. 3). Clade 3 also contained two Thai sub-clades, one of which was composed of clinical isolates. The second sub-clade consisted largely of environmental isolates but had recently diverged from a clinical Thai isolate (Fig. 3). Global isolates in clade 3 were of hospital origin and nosocomial infections.

**Fig. 4:**
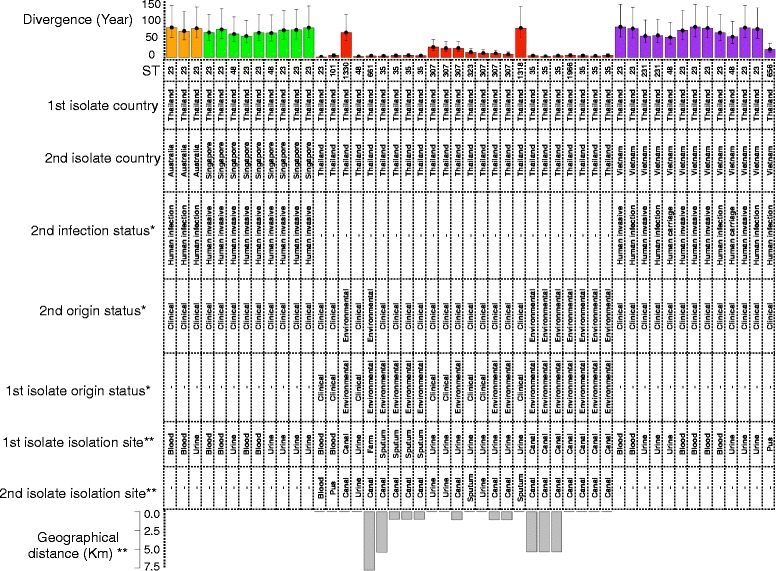
Origin, sample type, and isolate features for divergences between a Thai isolate and any other isolate from the Thai or global collection for non-clade 1 and 3 isolates over the past 50 years. This time corresponds to the formation of clade 1. To obtain the age and the upper and lower bounds for the 95% confidence interval, we divided the SNP distance by the mean substitution rates of clades 1 and 3 and the means of the lower and upper values for the 95 confidence intervals of the substitution rates estimated for clades 1 and 3. Each *color* corresponds to one country. The symbols ** and * signify the specific features of the Thai and the global collection, respectively. The *lower bar plot* shows geographical distance for Thai isolate pairs

To generalize our findings about the epidemiological links between global and clinical and environmental Thai isolates for the isolates not included in clades 1 or 3, we excluded the isolates in the two clades and then used the average substitution rates for isolates in clades 1 and 3 to estimate divergence times between a Thai isolate and any other Thai or global isolate that was less than 250 SNPs apart (this corresponds to approximately 50 years, which is the age of the MRCA of clade 3, which is older than clade 1). This revealed that Thai isolates appeared to have diverged more recently from each other than from the global isolates (Fig. 4). The majority of recent divergences in the Thai collection occurred between isolates from the same origin of isolation, i.e., environment or hospital (17 out of 24). However, there were several cases of recent divergence involving isolates of both environmental and hospital origin, as well as evidence for recent divergence between isolates from different environmental origins, i.e., different canals (Fig. 4). Moreover, one putative transmission event may have occurred between isolates of canal and farm origin (Fig. 4). The Thai isolates involved in 25 recent divergence events between a Thai and global isolates were all of clinical origin, and 15 and 7 of these isolates were recovered from invasive and non-invasive infections, respectively (Fig. 3). Furthermore, 19 out of 25 cases occurred between ST23 isolates, a well-known hyper-virulent strain. Taken together, these findings indicate that *K. pneumoniae* has rapidly expanded within the environment and hospitals, and that in some instances, isolates of different origins have only recently diverged. Furthermore, Thai isolates were closely related to the clinical sub-population of the global collection, suggesting that the clinical collection is a part of a global circulation of hyper-virulent *K. pneumoniae* (Fig. 3).

We then investigated the distribution of plasmid replicons and virulence factors in the Thai isolates. The predominant plasmid replicons were KpN3 and ColMG828 and less frequently the R plasmid, all of which are known to carry multiple resistance and virulence genes (Additional file 2: Figure S1). The majority of the global *K. pneumoniae* isolates also harbored these plasmid replicons (results not shown), indicating their global distribution. These replicons were present in isolates from both the environment and clinical samples (Additional file 2: Figure S1). Of the 75 virulence factors considered, 10 were present in >95% of isolates, examples being the *mrk* genes encoding fimbrial biosynthesis proteins and *iutA* encoding ferric aerobactin receptor [32]. By contrast, more than 40% of virulence genes were exclusively present in the clinical isolate collection (Additional file 2: Figure S2), including genes involved in capsule synthesis (*rmpA*) [33], iron transport (*iro*), phospholipid transport (*mce*), regulation and transport (multiple *clb* genes), and transcription regulation (*kvgA*). Multiple iron metabolism-related and siderophore genes including *ybt*, *irp*, and *fyuA* were more common (statistical significance level for z-statistic of logistic regression: *p* value < 0.05) in clinical isolates and were incorporated into the chromosome. The higher number of virulence factors in clinical versus environmental isolates (Additional file 2: Figure S2), especially those involved in iron uptake, is suggestive of hyper-mucoviscous and hyper-virulent strains that may be more efficient in iron uptake and capsule production [34]. Some virulence genes were integrated into the chromosome while others were plasmid-mediated, implying that multiple mechanisms mediate their acquisition. Some virulence genes were also present in the genome of other gram-negative bacteria such as *E. coli* and *Citrobacter koseri*, indicating sharing both within and between species. A full list of the virulence factors with their frequencies in clinical and environmental isolates is provided in Additional file 3.

With the exception of carbapenems, trimethoprim, tigecycline, and aminoglycosides, the Thai isolates exhibited intermediate to high resistance levels to the antibiotics tested. This finding, along with a correlation between increased minimum inhibitory concentration (MIC) values and antibiotics with a similar mechanism of action, is indicative of a limited number of effective antibiotics to treat infections (Additional file 2: Figure S3 and Figure S4). All isolates were ESBL-positive based on phenotypic testing. A genome-wide screen for known ESBLs as defined by the Comprehensive Antibiotic Resistance Database (https://card.mcmaster.ca/) demonstrated that *bla*_CTX-M-15_ *, bla*_SHV_ *, bla*_*VEB*_, and *bla*_GES_ accounted for the ESBL phenotypes in the population. *bla*_GES_ and *bla*_VEB_ gene copies were found exclusively in the environmental hospital sewer isolates and clinical isolates, respectively. By contrast, *bla*_CTX-M-15_ and *bla*_SHV_ had been sporadically gained by both environmental and clinical isolates. Nineteen isolates (25%) were found to be multidrug-resistant (resistant to three or more drug classes), all of which were clinical isolates. Four isolates were resistant to the carbapenem drugs. Resistance in these isolates was associated with the presence of plasmid-associated genes encoding carbapenamases, specifically New Delhi metallo-β-lactamase (NDM) (in two multidrug-resistant clinical isolates) and GES (in two non-multidrug-resistant isolates from the hospital sewer). These genes appear to have been acquired in different lineages across the phylogenetic tree (Additional file 2: Figures S5 and S6). Isolation of *Klebsiella oxytoca* and *Enterobacter cloacae* from hospital sewers that harbor GES has been reported previously [35] and indirectly reflects its presence in the hospital population or environment. Additional file 4 provides a list of β-lactamases and ESBLs in the collection.

Our results indicated that isolates with an increase in MIC values and resistant phenotypes occurred in different lineages throughout the phylogenetic tree (Additional file 2: Figure S4). However, the MIC values for β-lactams (especially cephalosporins and aztreonam) were significantly higher in clinical isolates compared with environment isolates (Additional file 2: Figure S5). Consistent with this, clinical isolates carried more copies of β-lactamase genes (Additional file 2: Figure S6). Besides the chromosomally encoded *bla*_LEN_, *bla*_SHV_, and *bla*_OKP_ that were present in every isolate, *bla*_OXA_, *bla*_TEM_, and *bla*_CTX_ occurred throughout the tree, and these genes, in combination with other β-lactamases, were more common in clinical isolates (Additional file 2: Figure S6), which is consistent with reports from numerous countries.

No patterns were observed in the presence/absence of resistance genes for the non-β-lactam antibiotics in environmental versus hospital isolates (Additional file 2: Figure S6). The *oqx* efflux pump gene was present in every isolate and was not correlated with ciprofloxacin resistance. However, non-synonymous SNP densities within DNA topoisomerase IV genes (*parE* and *parC*) and point mutations in D87 DNA gyrase A, as well as in the E84 DNA topoisomerase IV genes, were exclusively present in two isolates with high MIC values for ciprofloxacin. Both mutations occurred in the quinolone resistance-determining region (QRDR) of *gyrA* and *parC* [36, 37]. These point mutations were only found in clinical isolates, although both have been detected previously in *E. coli* isolated from aquatic environments [38]. The aminoglycoside resistance genes were noted to have been acquired on multiple occasions throughout the phylogenetic tree. Even though *K. pneumoniae* is reported to be intrinsically resistant to tetracyclines, the acquisition of further copies of tetracycline resistance genes occurred across the tree in both clinical and environmental isolates (Additional file 2: Figure S6).

## Discussion

In this study we investigated the epidemiology of *K. pneumoniae* in a defined geographic area that included a general hospital and surrounding canals and farms. Our findings support the suggestion that clinical *K. pneumoniae* have evolved mechanisms to better adapt to survival in the clinical setting. Virulence factors were more frequent in clinical isolates and had been acquired on more than one occasion. This indicates the selection and dissemination of virulent strains in hospitals. Furthermore, the higher number of β-lactam resistance genes in clinical isolates together with higher absolute MIC values for some β-lactams for *K. pneumoniae* of clinical origin can be attributed to higher exposure to antibiotics, as proposed previously [39]. Given our finding of recent divergence events between clinical and environmental isolates, our results suggest that the selective pressure imposed upon clinical isolates is sufficient to result in significant changes in the genome of clinical isolates within a few decades. It has been reported previously that the prevalence of antibiotic resistance of food-borne isolates is higher for meat-source *K. pneumoniae* isolates than for human clinical isolates [16]. However, due to the lack of sufficient data about the strength of selective antibiotic pressure in food-animal production versus hospitals, it is not possible to draw a definitive conclusion about antibiotic use and resistance [16].

The detection of carbapenem-resistant isolates in pre-treated hospital wastewater reiterates the importance of the treatment of hospital wastewater prior to release into the environment [40, 41]. Our study also highlights the role of environmental water as a potential reservoir for *K. pneumoniae*, where antibiotic resistant isolates may emerge. Antibiotic resistance genes present in environmental isolates were present in the founder lineage in some cases, for instance, in isolates in the mixed environmental and clinical clade 3 (Fig. 3 and Additional file 2: Figure S3), which is consistent with contamination of such reservoirs. Resistance was also noted to have emerged in some environmental lineages against various antibiotics, for instance, ertapenem, cefoxitin, amikacin, and amoxicillin-clavulanic acid in sewage water isolates (Additional file 2: Figure S3), which presumably arises in response to antibiotics in environmental wastewater, as reported previously [42, 43]. Several studies have shown the presence of highly antibiotic-resistant bacteria and resistance genes in sewage released into aquatic environments [42, 44–46]. In line with these findings, our results suggest that the release of untreated hospital sewage may play a role in the environmental emergence and spread of multiresistant pathogenic bacteria, and that wastewater (including hospital waste) warrants treatment to eliminate these organisms prior to release. This may be of particular importance in low-income rural areas and countries, where people are in greater contact with wastewater and may consume contaminated food and water containing high levels of antibiotic-resistant bacteria. Of note, wastewater is treated prior to release from Bhuddhasothorn Hospital, and no ESBL-positive *K. pneumoniae* were isolated from post-treated waste.

## Conclusions

In this study the availability of epidemiologic information and the high resolution of whole genome sequencing allowed us to discover epidemiologic links between clinical and non-clinical *K. pneumoniae*. Limitations of the collection were the sparseness of the environmental collection and the lack of non-ESBL-producing strains. This happened because our isolates were selected using media that were selective for ESBL production. The isolation of *K. pneumoniae* from highly contaminated samples is challenging without the use of selective culture, but our approach means that the environmental collection was biased towards clinically important isolates, i.e., ESBL-producing strains. This facilitated the identification of distinct genomic patterns relating to the distribution of antimicrobial resistance and virulence factor genes in clinical isolates, but the inclusion of non-ESBL isolates, particularly from environmental sites, is required to obtain a broader assessment of clinical and non-clinical populations. The inclusion of susceptible isolates from the environment and hospital may reduce the difference in antibiotic resistance observed here between clinical and environmental isolates, although the effect on virulence gene pattern is difficult to predict. Future studies based on larger and deeper collections will be required to gain a detailed understanding of global transmission of *K. pneumoniae* at different geographical scales.

## Additional files

Additional file 1: Table S1. Supplemental Table S1, which includes accession numbers and the metadata for the studies on isolates here. (CSV 33 kb)
Additional file 2: Figures S1–S6. Figures and descriptions of Supplemental Figures S1–S6 mentioned throughout the manuscript. (DOCX 1927 kb)
Additional file 3: Table S2. Supplemental Table S2, which includes the list of putative virulence factor genes. (CSV 9 kb)
Additional file 4: Table S3. Supplemental Table S3, which includes the list of β-lactamase genes and ESBLs. (CSV 5 kb)

## Abbreviations

CI: Confidence interval
CLSI: Clinical and Laboratory Standards Institute
ENA: European Nucleotide Archive
ESBL: Extended-spectrum β-lactamase
*K. pneumoniae*: *Klebsiella pneumoniae*
MIC: Minimum inhibitory concentration
MLST: multilocus sequence typing
MS: Mass spectrometry
