# Supplementary material for: Whole genome sequencing reveals high-resolution epidemiological links between clinical and environmental Klebsiella pneumoniae
Source: Genome Med. 2017 Jan 24;9:6. doi: 10.1186/s13073-017-0397-1 (PMC5264300; doi:10.1186/s13073-017-0397-1)
Supplement: Additional file 2: Figures S1–S6. — Figures and descriptions of Supplemental Figures S1–S6 mentioned throughout the manuscript. (DOCX 1927 kb) [file 13073_2017_397_MOESM2_ESM.docx]

**SUPPLEMENTARY FIGURES**

**Whole genome sequencing reveals high-resolution epidemiological links between clinical and environmental *Klebsiella pneumoniae***

Chakkaphan Runcharoen^*^, Danesh Moradigaravand^*^, Beth Blane, Suporn Paksanont, Jeeranan Thammachote, Suthatip Anun, Julian Parkhill, Narisara Chantratita^**^ and Sharon J. Peacock^**^

* Joint first author

** Joint senior author

Figure S1 Distribution of plasmid replicons across the phylogenetic tree. The barplots show the relative frequency of each gene in the clinical (green) and environmental (red) sub-populations for each plasmid.

Figure S2 Distribution of virulence factors across the phylogenetic tree. The boxplots show the number of virulence genes within the clinical and environmental sub-populations. The symbol *** denotes that the difference between the two was significant at p-value <10^-3^ (obtained from Student’s t-test, testing mean difference). The barplots show the relative frequency of each gene in the clinical (green) and environmental (red) sub-populations for each gene.

Figure S3 Correlogram of MIC values for different antibiotics. The blue and red colors correspond to positive and negative correlations, respectively. B) The resistance pattern for antibiotics tested in this study.

Figure S4 Distribution of MIC values across the phylogenetic tree. The first column shows the isolation origin of the isolate. B) The distribution of resistance/intermediate/susceptible phenotypes across the phylogenetic tree.

Figure S5 MIC distributions for different antibiotics in the environmental and clinical sub-population. The symbols ** and *** denote that the difference between the two was significant at p-values 10^-2^ and 10^-3^, respectively (obtained from Student’s t-test, testing mean difference).

Figure S6 Distribution of antibiotic resistance genes across the phylogenetic tree. The boxplot show the number of antibiotic resistance genes within the clinical and environmental sub-populations. Groups 1, 2, 3, 4, 5, 6 and 7 correspond to beta-lactamases, fluoroquinolone, tetracycline, sulphonamides, aminoglycosides and other antibiotics resistance genes, respectively. The barplots show the relative frequency of each gene in the clinical (green) and environmental (red) sub-populations for each gene. R.F. and AMR in the figure stand for relative frequency and antimicrobial resistance, respectively.
